# Supplementary material for: Sesquiterpene Lactones as Promising Anti-Glioblastoma Drug Candidates Exerting Complex Effects on Glioblastoma Cell Viability and Proneural–Mesenchymal Transition
Source: Biomedicines. 2025 Jan 8;13(1):133. doi: 10.3390/biomedicines13010133 (PMC11761231; doi:10.3390/biomedicines13010133)
Supplement: Supplementary file 1 [file biomedicines-13-00133-s001.zip › Supplementary_Materials.pdf]

Review

Sesquiterpene lactones as promising anti-glioblastoma drug candidates exerting complex effects on glioblastoma cell viability and proneural-mesenchymal transition

Andrey V. Markov *, Arseny D. Moralev and Kirill V. Odarenko

Institute of Chemical Biology and Fundamental Medicine, Siberian Branch of the Russian Academy of Sciences, Lavrent'ev Avenue 8, 630090 Novosibirsk, Russia; arseniimoralev@gmail.com (A.D.M.); k.odarenko@yandex.ru (K.V.O.)

* Correspondence: andmrkv@gmail.com or markov_av@niboch.nsc.ru

Supplementary Materials

Analysis of the association of primary protein targets of STLs with glioblastoma regulome

The list of genes associated with the proneural and mesenchymal subtypes of glioblastoma was obtained from the Molecular Signatures Database (MSigDB) [1,2]. Subsequently, the gene association network was reconstructed using the STRING database (confidence score > 0.7) [3]. The experimentally verified protein targets of STLs identified from published data were integrated into the glioblastoma-related gene network, after which the maximum clique centrality score (MCC) was calculated for each STLs target using the cytoHubba plugin [4] in Cytoscape 3.9.1 software to determine their involvement in the glioblastoma regulome.

References

1. Liberzon, A.; Birger, C.; Thorvaldsdóttir, H.; Ghandi, M.; Mesirov, J.P.; Tamayo, P. The Molecular Signatures Database Hallmark Gene Set Collection. *Cell Syst.* **2015**, *1*, doi:10.1016/j.cels.2015.12.004.

2. Verhaak, R.G.W.; Hoadley, K.A.; Purdom, E.; Wang, V.; Qi, Y.; Wilkerson, M.D.; Miller, C.R.; Ding, L.; Golub, T.; Mesirov, J.P.; et al. Integrated genomic analysis identifies clinically relevant subtypes of glioblastoma characterized by abnormalities in PDGFRA, IDH1, EGFR, and NF1. *Cancer Cell* **2010**, *17*, 98–110, doi:10.1016/j.ccr.2009.12.020.

3. Szklarczyk, D.; Franceschini, A.; Wyder, S.; Forslund, K.; Heller, D.; Huerta-Cepas, J.; Simonovic, M.; Roth, A.; Santos, A.; Tsafou, K.P.; et al. STRING v10: Protein-protein interaction networks, integrated over the tree of life. *Nucleic Acids Res.* **2015**, *43*, D447–D452, doi:10.1093/nar/gku1003.

4. Chin, C.H.; Chen, S.H.; Wu, H.H.; Ho, C.W.; Ko, M.T.; Lin, C.Y. cytoHubba: Identifying hub objects and sub-networks from complex interactome. *BMC Syst. Biol.* **2014**, *8*, doi:10.1186/1752-0509-8-S4-S11.
